# Publication ethics – What do we need to know?

**DOI:** 10.1002/acm2.12570

**Published:** 2019-03-20

**Authors:** Michael Mills

A sweeping and comprehensive revision to the AAPM Code of Ethics was established and approved by the AAPM Board of Directors on 28 November 2018. The document was published in *Medical Physics* on 20 December 2018, and became effective 1 January 2019. This document may be viewed here: https://aapm.onlinelibrary.wiley.com/doi/full/10.1002/mp.13351.

This editorial will review only the “Publication Ethics” section; however, all readers are encouraged to review the document in its entirety. The Code of Ethics has been written to serve the broader purpose of giving practical and ethical guidance to AAPM members for making proper decisions in their professional lives. It also contains a section that details how ethical complaints will be processed.

The following excerpt is from Section 3. Guidelines; III. Research Ethics; D. Publication Ethics. I will discuss this section as it pertains to publication of archival research, clinical, and professional articles within the JACMP.

## D. PUBLICATION ETHICS

Members who find themselves involved in any aspect of publishing (commercial, newsletter, editorial, or academic; as authors, reviewers, or editors) are expected to represent themselves and their subject matter with honesty and transparency. When Members are listing their published work, transparency requires disclosure of the existence and nature of the review process for the published work.

### Authorship

- Members must adhere to the requirements of the publication to which they are submitting. Members should reserve authorship only for those who:
- have contributed substantially to the conception and design, and/or acquisition of data, and/or analysis and interpretation of data;
- were directly involved in the drafting and/or revising of the publication; and
- have given final approval of the version to be submitted for review.
- Members must not plagiarize the work of others.
- Members must not self‐plagiarize, or submit for publication with substantially similar material to two or more journals, unless the manuscript was rejected or the editors of all involved journals grant permission.
- Members should respect the peer‐review process by considering the concerns raised by previous reviewers before resubmitting their manuscript to another journal.

### Declaration of interests

- Members must explicitly declare all financial interests with respect to business or corporate entities when submitting manuscripts or giving presentations, even if such arrangements are tangential to the subject matter of the work. Such financial interests may include sponsorship, travel reimbursement, performance‐based bonus incentives, or stock ownership.

### Editorship and peer review

- Members acting as editors or reviewers:
- should be aware of potential bias or conflict of interest and strive to deliver an impartial assessment of the work based on merit alone;
- must declare and manage any conflicts of interest that could compromise their objectivity;
- should ensure that the peer‐review process is objective, fair, and confidential;
- are responsible for maintaining the dialogue, and any communication among participants, at a professional and respectful level throughout the review process;
- must not use the unpublished results to benefit their own work or advancement; and
- must not prevent publication of results in order to benefit their own work or advancement.
- Members acting as editors of non‐peer‐reviewed publications must not knowingly publish falsified or plagiarized data.

I will review each of these four sections and provide insights based on the experiences of the JACMP editorial team.

Introductory paragraph, “D. Publication ethics” — You may be an author, reviewer, or editor. Regardless of your role, if there is a nagging issue respecting credit, responsibility, accountability, process, or interpretation of an article, you share responsibility. Others are watching, and what may be a small issue to you is more than likely a large issue to someone else. If you have a potential conflict, do not avoid the issue, nor should you seek out your best friend for an opinion. If you are an author or reviewer, disclose the issue to the editor; if you are an editor, take the discussion to the Editor‐in‐Chief team.

#### “a. Authorship”

Some types of authorship are not allowed. No one should receive “courtesy or honorary authorship” for reviewing the article in or providing secretarial support. Similarly, “gift authorship” to thank someone for reciprocal author placement or for some other favor is not appropriate. Senior figures should not be included in an author list for the purpose of drawing attention to the paper, nor should they use their influence to direct the inclusion of any author who did not meet the criteria for authorship.

Plagiarism and other forms of publication misconduct can take on several forms, some of them subtle. Splitting one study into two is an example. Follow‐up publications with no new conclusions, or publishing similar studies in different journals is another example. Finding an obscure unpublished thesis or dissertation and publishing it under your own name is an obvious example of plagiarism. However, simply failing to properly acknowledge a source could also be plagiarism. It is even possible to plagiarize yourself by quoting large sections of already published content without a proper reference.

#### “b. Declaration of interests”

The underlying reason for disclosing relationships and value exchanges is to acknowledge the potential for bias or conflict of interest. The onus is usually on the editorial team to decide whether the conflict between an author's duty to publication ideals is subverted by personal benefits. This is usually difficult to do even when there is full disclosure of potential interests and bias. It is not just the author, but also reviewers who may be biased for any number of reasons. We depend on both authors and reviewers to be forthcoming whenever such biases may exist so any potential conflicts may be managed.

#### “c. Editorship and peer review”

The JACMP adopted a double‐blind peer‐review model at its inception, back in the winter of 2000. A double‐blind model helps manage potential conflicts and retributions between authors and reviewers. Additionally, a double‐blind review is thought by many to be a desirable model attribute to provide objective and fair reviews for all submissions. We rely on our reviewers to disclose potential conflicts, or if they are aware of the identity of the author. Additionally, if a reviewer thinks a conflict may exist, that individual is expected to ask to be recused. Reviewer comments are not published and this is a protection both for the reviewers and authors.

Finally, I want to call to the reader's attention the existence of AAPM Policy PP‐16‐C, Medical Physics and JACMP Policies and Procedures for Reviewing and Adjudicating Individual Cases of Alleged Violations of Standards for Scientific Integrity and Conduct, found here: https://www.aapm.org/org/policies/details.asp?id=206&type=PP&current=true.

This document became effective on 25 November 2018, and has a sunset date of 31 December 2023. This report was drafted by the Scientific Integrity Subcommittee (SISC) of the Medical Physics Editorial Board, whose membership was expanded in 2018 to include representation from JACMP leadership. Although this document is very detailed and intended to address most anticipated aspects of ethical behavior respecting publications, it should be read by every editor, associate editor, and every individual responsible for publications affiliated with the AAPM.

Remember, when in doubt: assess, assume responsibility, disclose and, if necessary, recuse!

